# The Anti-Cancer Activities of *Vernonia amygdalina* Extract in Human Breast Cancer Cell Lines Are Mediated through Caspase-Dependent and p53-Independent Pathways

**DOI:** 10.1371/journal.pone.0078021

**Published:** 2013-10-24

**Authors:** Fang Cheng Wong, Chern Chiuh Woo, Annie Hsu, Benny Kwong Huat Tan

**Affiliations:** Department of Pharmacology, Yong Loo Lin School of Medicine, National University of Singapore, Singapore; Stony Brook University, United States of America

## Abstract

Breast cancer is currently the leading cause of cancer-related deaths among women globally. Notably, medicinal plant extracts may be a potential source for treatments of breast cancer. *Vernonia amygdalina* (VA) is a woody shrub reported to have not only diverse therapeutic effects but also anti-cancer properties. However, current research about the mechanisms of the anti-cancer potential of VA has been limited. This study aimed to investigate the mechanisms of action of VA that underlie its anti-cancer effects in human breast cancer cell lines (MCF-7 and MDA-MB-231 cells). Results from MTT assay revealed that VA inhibits the proliferation of MCF-7 and MDA-MB-231, in a time- and dose-dependent manner. The underlying mechanism of this growth inhibition involved the stimulation of cell-type specific G1/S phase cell cycle arrest in only MCF-7 cells, and not in MDA-MB-231 cells. While the growth arrest was associated with increased levels of p53 and p21, and a concomitant decrease in the levels of cyclin D1 and cyclin E, it was shown that VA causes cell cycle arrest through a p53-independent pathway as tested by the wild type p53 inhibitor, pifithrin-α. Furthermore, this study revealed that VA induces apoptosis in the two cell lines, as indicated by the increase in Annexin V-positive cells and sub-G1 population, and that this VA-induced apoptosis occurred through both extrinsic and intrinsic apoptotic pathways. The apoptosis in MCF-7 cells was also likely to be caspase-dependent and not p53 transcriptional-dependent. Given that approximately 70% of diagnosed breast cancers express ER-α, a crucial finding was that VA inhibits the expression of ER-α and its downstream player, Akt, highlighting the potential clinical significance of VA. Moreover, VA exhibits synergism when combined with doxorubicin, suggesting that it can complement current chemotherapy. Overall, this study demonstrates the potential applications of VA as an anti-cancer drug for breast cancer treatment.

## Introduction

As the most common cancer among women around the globe, breast cancer is currently the leading cause of cancer-related deaths in the world [1], [2]. The search for new and effective drugs to treat breast cancer is thus of central importance, given the drawbacks involved in current treatment methods that compromise their effectiveness. In this search for new drugs, medicinal plant extracts may be a potential source for alternative novel treatments of breast cancer. Notably, there has been growing interest in one particular plant as a source for anti-cancer drugs due to its diverse medicinal uses in traditional folk medicines. *Vernonia amygdalina* (VA) is a woody shrub that can grow up to 5 m tall, belonging to the family of Asteraceae [3]. Native to Nigeria (West Africa) and widely grown in Africa [3], VA is also found in Asia, and is especially common in Singapore and Malaysia [4], [5]. The leaves exhibit a characteristic odor and bitter taste, explaining its common English name of ‘bitter leaf’. Owing to the lack of documentation of this plant, different local names have been used in various countries, such as Etidod or Ewuro in Nigeria, South African leaf in Malaysia and Kenya, as well as Non-tree South in China [5]. The medicinal properties of the plant, however, are widely recognized. As one of the plants that forms a major portion of the naturalist's pharmacopeia in Nigeria [6], VA has been shown to possess diverse therapeutic effects such as anti-malarial, anti-microbial (anti-bacterial, anti-fungal, anti-plasmodial, etc.), anti-diabetic and anti-cancer effects [5], [7]. The anti-cancer effect of VA was first shown in human carcinoma of nasopharynx and later in leukemia cells P-388 and L-1210 using the chloroform extract of VA [8], [9]. Different extracts of VA have thus been used in scientific research to reveal the therapeutic properties of this plant. Current research conducted on the anti-cancer effect of VA has focused exclusively on MCF-7 cells, and as Ijeh *et al*. [7] recognize, a significant proportion of studies on VA has been haphazard and peripheral. Thus, further research is needed to elucidate the specific mechanism of actions systematically, in order to provide crucial insights about the treatment of breast cancer. Moreover, there are other types of breast cancer cells, of which the claudin-low or triple-negative subtype is known to be the most aggressive with poor prognosis. It is important to take into account the different subgroups of breast cancer types and recognize the differential responses even within subgroups when screening potential new breast cancer therapies.

This study hypothesized that VA exerts its anti-cancer effects through the induction of cell cycle arrest and apoptosis on human breast cancer cells. Based on this hypothesis, the study aimed to investigate the anti-cancer effects of VA and its possible mechanisms of action in two human breast cancer cell lines, namely MCF-7 and MDA-MB-231.

## Materials and Methods

### Ethic statement

The leaves of *Vernonia amygdalina* were collected with the owner's permission from a private farm owned by Mr. Ong Ko and Mrs. Hoi Soi Chan.

### Preparation of plant extract

Fresh leaves of *Vernonia amygdalina* were collected from a private farm in Johor, Malaysia. Harvested fresh leaves (151.1 g) were rinsed with distilled water and soaked in 80% denatured ethanol (2 L). The leaves were blended with homogenizer (Wiggen Hauser D-500) and the mixture was left for 1 hour. The extracted mixture was then filtered using 0.9 mm filter membrane by vaccum pump (Gast USA DOA-PIO4-BN). The filtrate was concentrated in a rotary evaporator at 50°C (Buchi Labortechnik AG, Postfach, Switzerland). The resultant dark green condensate was subjected to freeze-drying (Christ Gamma 1-16 LSC) for 1–2 days and the resultant solid residue (9.58 g of dark green powder) is referred to as ‘extract’ in this paper. The extract yield (w/w) from 151.1 g of fresh VA leaves was approximately 6.32%. The extract was prepared fresh in 10% dimethyl sulfoxide (DMSO) prior to usage.

### Cell lines

Two human breast cancerous cells lines, MCF-7 and MDA-MB-231, were purchased from American Type Culture Collection (ATCC) (Manassa, VA, USA). There are characteristic differences between MCF-7 and MDA-MB-231 cells: MCF-7 cells express estrogen receptor and wild type p53, but lack caspase-3 due to the deletion of CASP-3 gene; MDA-MB-231 cells are estrogen receptor-negative and express mutant p53 and functional caspase-3. The cells were cultured as monolayers in RPMI 1640 supplemented with 10% fetal bovine serum (FBS) and 1% antibiotic-antimycotic (ABAM) at 37°C in a humidified incubator of 95% air and 5% CO_2_.

### Reagents

ABAM, trypsin, propidium iodide (PI), annexin V-FITC, ribonuclease A (RNase A) from bovine pancreas, trizma base, glycine, ammonium persulfate (APS), NaCl, hepes, ethylene glycol tetraacetic acid (EGTA), ethylenediaminetetraacetic acid (EDTA), 100% triton-X, trypan blue, albumin, mercaptoethanol-6, RPMI 1640 powder, pifithrin-α, doxorubicin and 3-(4,5-Dimethylthiazol-2-yl)-2,5-diphenyltetrazolium bromide (MTT formazan) were purchased from Sigma-Aldrich (St. Louis, MO, USA). Z-VAD-fmk was purchased from Zuellig Pharma Pte. Ltd. (Singapore). Laemmli sample buffer, 40% acrylamide, Bradford protein assay kit, TEMED and western blotting membrane (0.45 µm) were obtained from Bio-Rad (Hercules, CA, USA). FBS was purchased from Hyclone (Loughborough, UK) whereas DMSO was purchased from MP Biomedicals (Solon, OH, USA). Sodium dodecyl sulfate (SDS) and phosphate buffered saline (PBS) were purchased from Vivantis Technologies (Selangor, Malaysia). Low fat milk powder (Anlene) was purchased from Fairprice supermarket (Singapore) while tween 20 was purchased from Merck & Co., Inc. Enhanced chemiluminescence select (ECL) was obtained from GE Healthcare (Little Chalfont, Buckinghamshire, UK). Antibodies such as PARP, p53, Bcl-2, Bcl-xL, Bax, Bak, p21, cyclin D1, caspase-7, caspase-9, ER-α, p-Akt (Thr308), p-GSK3β (Tyr216), total GSK3β, chicken anti-rabbit IgG horse-radish peroxidase-linked and chicken anti-mouse IgG horse-radish peroxidase-linked antibodies were purchased from Santa Cruz Biotechnology (Santa Cruz, CA, USA). Cyclin B1, cyclin E, caspase-8, caspase-3, cleaved caspase-3 and β-actin were purchased from Cell Signaling Technology (Beverly, MA, USA).

### Cell proliferation analysis

The anti-proliferative effects of VA on MCF-7 and MDA-MB-231 cells were examined using MTT colorimetric assay. Cells were seeded in 96-well plates at a density of 5×10^3^ cells per well for 24 h before exposure to the indicated concentrations of VA for 24, 48 and 72 h respectively. 0.5% DMSO was used as a negative control (0 µg/ml). MTT reagent was dissolved at a concentration of 5 mg/ml in sterile PBS at room temperature. After removal of the medium, 20 µl was added to each well and followed by 4 h incubation. The MTT solution was carefully aspirated and the purple formazan crystals produced by the mitochondrial dehydrogenase enzymes were dissolved in DMSO. The optical density (OD) of each well was measured at 570 nm on a scanning multiwall spectrophotometer (TECAN infinite M200, Mannedorf, Switzerland).

### Flow cytometric cell cycle analysis

The effect of VA on MCF-7 and MDA-MB-231 cell cycle distribution was determined by flow cytometric analysis. Cells were seeded in six-well plates at 1.4×10^5^ cells per well, cultured for 24 h and synchronized by serum deprivation for overnight prior to the indicated concentrations of VA exposure for 24, 48 and 72 h respectively. The cells were detached from the plates by trypsinization and fixed in 70% cold ethanol (added in a drop-wise manner) for at least 2 h at 4°C. Prior to flow cytometric analysis, the cell solutions were centrifuged at 300g for 5 min and the pellet was re-suspended in 1 ml of PBS/1% FBS. After centrifuging at 300 g for 5 min, the fixed cells were then treated with 0.5 ml of RNase A (200 µg/ml) and incubated for 10 min at room temperature. The DNA content per cell was analyzed using flow cytometry (BD LSRFortessa™ cell analyzer, San Jose, CA, USA) after being stained with PI staining solution (2 µg/ml) for 20 min at room temperature in darkness. Offline analysis of cell cycle distribution was performed using Summit4.3 software (Beckman Coulter, Inc). Apoptotic cells with hypodiploid DNA content were measured by quantifying the sub-G1 peak in the cell cycle pattern. 10,000 events per sample were recorded for each experiment.

### Annexin V-FITC (fluorescein isothiocyanate) /PI (propidium iodide) assay

Apoptotic and necrotic cells were differentiated using the Annexin V-FITC kit according to the manufacturer's protocol (Miltenyi Biotec, Bergisch Gladbach, Germany). Cells were plated on six-well plates at 1.4×10^5^ cells per well and incubated for 24 h prior to the indicated concentrations of VA exposure for 24, 48 and 72 h respectively. The cells were harvested by trypsinization, washed with 1× Annexin V binding buffer, and re-suspended in 10 µl Annexin V-FITC-added binding buffer for 15 min in darkness. The stained cells were then washed with 1× Annexin V binding buffer and stained with 5 µl PI-added binding buffer immediately prior to analysis by flow cytometry (BD LSRFortessa Cell Analyzer, San Jose, CA, USA). 10,000 events per sample were recorded for each experiment.

### Western blot analysis

Cells were seeded in six-well plates at 2×10^5^ cells per well and followed by overnight incubation before treating with VA at the indicated concentrations and time intervals. Proteins from MCF-7 and MDA-MB-231 VA-treated cells were extracted with ice-cold cell lysis buffer (Cell signalling, Beverly, MA, USA) containing protease inhibitor cocktail (Calbiochem, Billerica, MA, USA). Protein lysates were centrifuged at 13,300 rpm for 5 min at 4°C to remove insoluble material; protein concentration in the supernatants was determined by the Bradford protein assay kit (Bio-rad Laboratories, Hercules, CA, USA) according to the manufacturer's instructions. A microplate reader (TECAN Infinite M200, Mannedorf, Switzerland) was used to measure the absorbance at 595 nm and the concentration of the protein was calculated based on a bovine serum albumin (BSA) standard curve with a range of 0 to 1 mg/ml. Protein lysates were boiled in Laemmli sample buffer (1∶1 dilution) (Bio-rad Laboratories, Hercules, CA, USA) at 100°C for 5 min and resolved by electrophoresis on 10% or 12% SDS polyacrylamide gels. After gel electrophoresis, the proteins were electrotransferred to a nitrocellulose membrane (0.45 µm) using Bio-Rad Trans-Blot SD Semi-Dry Electrophoretic Transfer Cell (Bio-rad Laboratories, Hercules, CA, USA) at 15 V for 28 min. Membranes were then blocked with 5% milk in TBS-Tween 20 for 30 min at 70 rpm, room temperature, followed by washing for 3×, 10 min each at 110 rpm. Thereafter, the membranes were probed with corresponding primary antibodies (1∶1000 dilution; e.g. PARP, p53, Bcl-2, Bcl-xL, Bax, Bak, p21, cyclin D1, caspase-7, caspase-9, ER-α, p-Akt (Thr308), p-GSK3β (Tyr216) and total GSK3β) for proteins of interest overnight at 4°C, 70 rpm. After overnight incubation, the membranes were washed 3×, 10 min each at 110 rpm before probing with corresponding secondary antibodies (chicken anti-rabbit or anti-mouse IgG conjugated with horse-radish peroxidase) diluted 1∶10000 in blocking buffer (Santa Cruz, CA, USA) for 1 hour at room temperature and followed by washing, 3×, 10 min each. The immunoblots were examined with the enhanced chemiluminescence kit (GE Healthcare, Little Chalfont, Buckinghamshire, UK). β-actin (Cell signalling, Beverly, MA, USA) was used in each blot as a loading control to ensure equal loading of proteins for each sample. Protein quantification was performed using ImageJ software (Java-based image processing program developed by National Institutes of Health).

### Evaluation of synergy between doxorubicin and VA

The Coefficient of Drug Interaction (CDI) was used to analyze synergistically inhibitory effects of drug combinations of doxorubicin and VA. CDI is calculated from the equation: CDI = AB ÷ (A×B), where AB is the ratio of the absorbance of the combination groups to control group; A or B is the ratio of the absorbance of the single agent group to control group. Hence, CDI values of <1,  = 1 or >1 would indicate that the drugs are synergistic, additive or antagonistic, respectively. In particular, CDI<0.7 indicates that the drugs are significantly synergistic [10].

### Statistical analysis

Results were analyzed by one-way analysis of variance (ANOVA) or Student's *t*-test, and differences were considered statistically significant at the level of p-values <0.05.

## Results

### VA inhibited cell proliferation in MCF-7 and MDA-MB-231 cells

**Figure 1:**
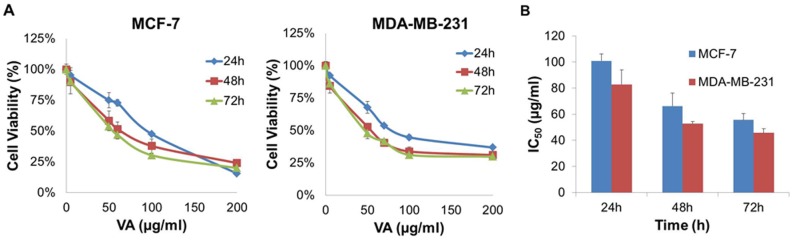
Anti-proliferative effect of VA on human breast cancer cells. (A) Dose-response curves of VA treatment in MCF-7 and MDA-MB-231 cells. Cells were cultured in 96-well plates and treated with indicated concentrations of VA (0–200 µg/ml) for 24, 48, and 72 h respectively. Cell viability was measured by MTT assay. Data represent the mean ± S.E.M. of three independent experiments. Statistical differences were analyzed with Student's t-test. (B) Comparison of IC_50_ of VA in MCF-7 and MDA-MB-231 cells at different time points. Values were derived from the graph of growth inhibition against drug concentration (µg/ml) from MTT assay. Data represent the mean ± S.E.M. of three independent experiments.

As a basis for further characterization of VA-induced cellular response in MCF-7 and MDA-MB-231, the ethanolic extract of VA was first examined for its ability to inhibit the proliferation of the two cell lines using MTT assay. As shown in **Figure 1A**, VA inhibited the proliferation of MCF-7 and MDA-MB-231 cells in a time- and dose-dependent manner. It was noticed that the cell viabilities of each cell line at 48 h and 72 h reflected minute differences, implying that the cells respond to VA within 48 h. Interestingly, at the highest dose of VA (200 µg/ml), cell viability of MCF-7 cells appeared to be independent of time (i.e. the drug effects are similar for each of the three indicated time points). Half-maximal inhibitory concentration (IC_50_) values are commonly used to evaluate the potency of a compound, in which the lower the IC_50_ value, the more potent the compound is. According to the results obtained from MTT assay, the IC_50_ values for MCF-7 cells were 100, 66 and 56 µg/ml at 24, 48 and 72 h respectively, whereas the IC_50_ values for MDA-MB-231 were 83, 53, 46 µg/ml at 24, 48 and 72 h respectively (**Figure 1B**). The ER-negative MDA-MB-231 cells were hence shown to be slightly more sensitive to VA-induced growth inhibition than MCF-7 cells. These results suggest that the cytotoxic action induced by VA may be independent of the estrogen receptor.

### VA induced G1/S cell cycle arrest in MCF-7 cells but not in MDA-MB-231 cells

**Figure 2:**
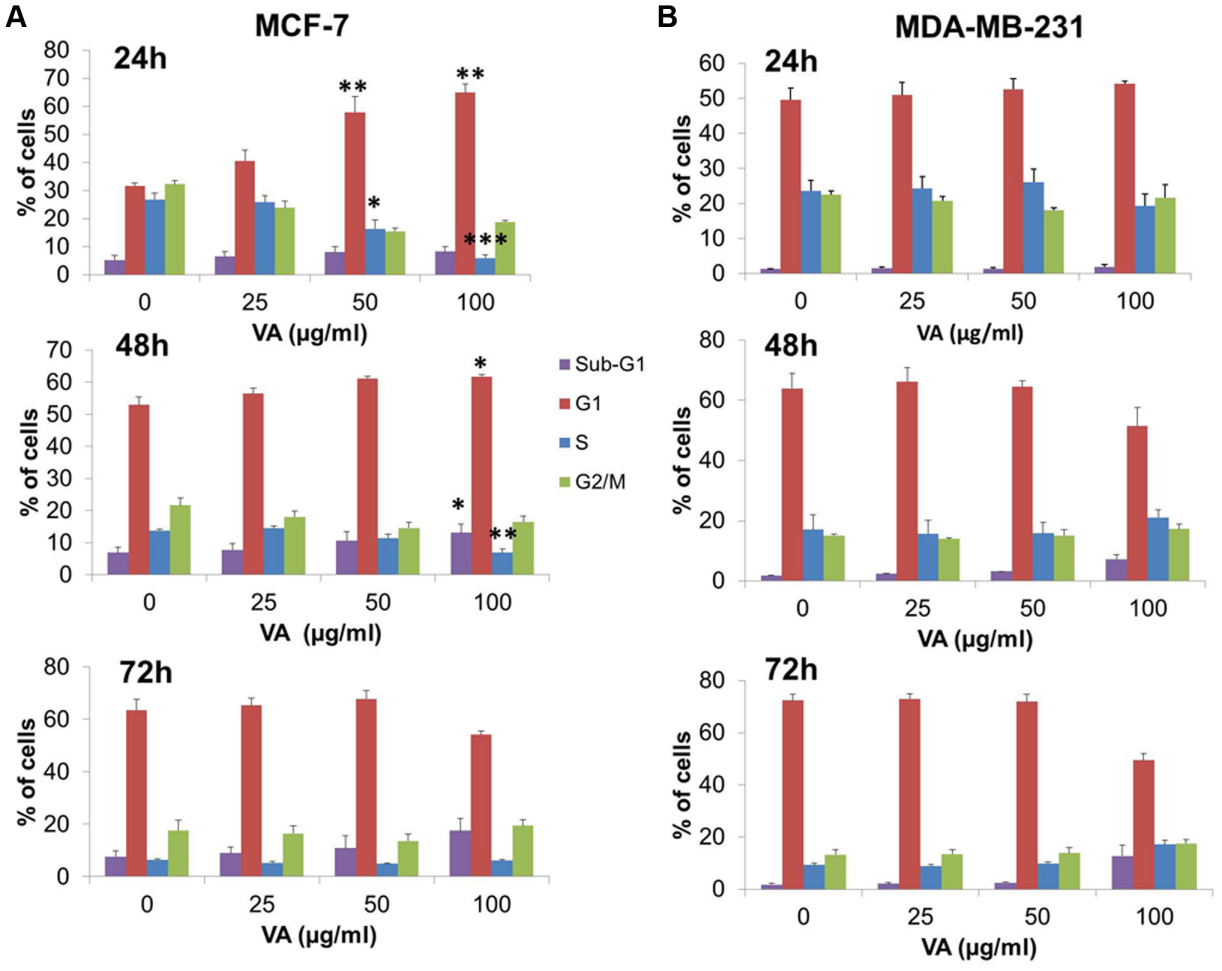
Changes in cell cycle phase distribution after VA treatment for 24, 48 and 72 Flow cytometric analysis of (A) MCF-7 cells and (B) MDA-MB-231 cells in different phases of the cell cycle according to VA concentrations. Cells were cultured and synchronized by serum-free medium for 24 h prior to VA treatment, and followed by staining with propidium iodide. Values shown are means ± S.E.M. of three independent experiments. Statistical differences were analyzed with one-way ANOVA test. *p<0.05, **p<0.01, ***p<0.001

Considering the fact that VA inhibited cell proliferation, flow cytometric analysis on cell cycle progression was performed to determine the mechanism for this anti-proliferative effect of VA on the breast cancer cells. In MCF-7 cells, VA induced time- and dose-dependent growth arrest in the G1 phase of the cell cycle, concomitant with a significant decrease in the S phase cells. For example, after exposure to an increasing dose of VA for 24 h, MCF-7 cells showed a marked increase by twofold, from 32% (control) to 65% (100 µg/ml) in the G1 phase, whereas the proportion of cells in the S phase decreased from 26% (control) to 6% (100 µg/ml) (**Figure 2A**). In terms of time-dependence, MCF-7 cells treated with 25 µg/ml VA demonstrated that after 24, 48 and 72 h exposure, the percentage of cells in G1 phase increased to 41%, 57%, and 65% respectively. Similarly, 50 µg/ml VA also caused G1 phase growth arrest across time, as shown by the increase in the percentage of cells to 58%, 61%, and 68% at 24, 48 and 72 h respectively. It is noteworthy that, following 100 µg/ml VA treatment, cells began accumulating in the sub-G1 phase at 48 h and the percentage of sub-G1 phase cells increased to 18% at 72 h (**Figure 2A**). This suggests that the cells may undergo apoptosis instead of cell cycle arrest because the damage induced by high concentration of VA was too severe to be repaired by DNA repair mechanisms within the cells. This observation could explain the reduction of cells in the G1 phase by 10% after the cells were treated with 100 µg/ml of VA for 72 h. Overall, these results indicated that VA-treated MCF-7 cells were arrested in the G1 phase of cell cycle at 24 h and 48 h, after which apoptosis occurred after exposure to the highest dose of VA for 72 h. It thus appears that VA is able to inhibit the growth of MCF-7 cells by promoting cell cycle arrest at the G1/S phase.

On the contrary, in MDA-MB-231 cells, the effect on cell progression after VA treatment was negligible, and no significant dose- or time-dependent growth arrest could be clearly observed. Increasing doses of VA did not result in the accumulation of G1 cells even after 72 h exposure (**Figure 2B**). However, following the highest dose of 100 µg/ml VA, cells accumulated in the sub-G1 phase, suggesting the occurrence of apoptosis in MDA-MB-231 cells. Taken together, VA caused growth arrest in the G1/S phase of the cell cycle only in MCF-7, but not in MDA-MB-231 cells, implying the differential regulation of VA on cell cycle progression between the two cell lines.

### VA-induced cell cycle arrest involved modulation of the expression of cell cycle regulators

**Figure 3:**
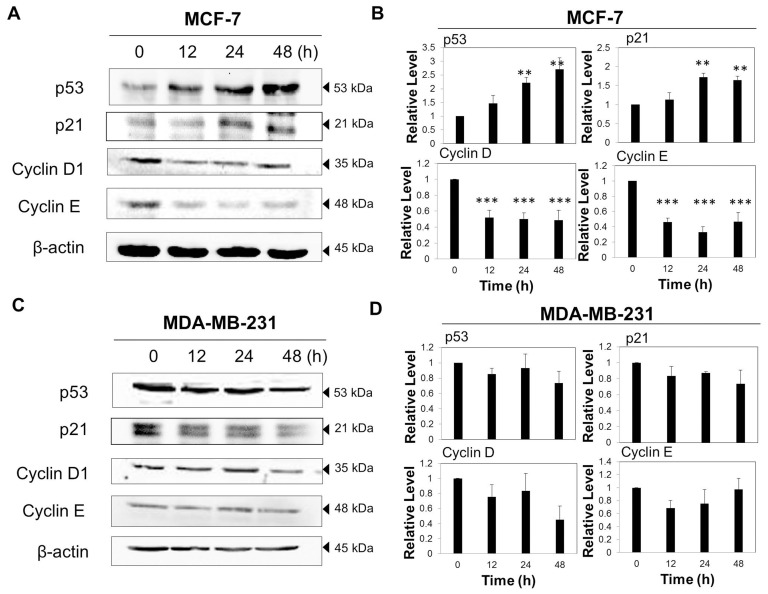
Effect of VA on the expression levels of p53, p21, cyclin D1 and cyclin E in MCF-7 and MDA-MB-231 cells. Time-dependent regulation by VA in (A) MCF-7 cells and (C) MDA-MB-231 cells. Cells were treated with VA (50 µg/ml) for 48 h. The data are representative of at least three independent experiments. (B) and (D) Protein quantification of the western blot results shown in (A) and (C) respectively. Protein levels were normalized to the β-actin level and are shown relative to the DMSO-treated control cells (normalized at 1). Statistical differences were analyzed with one-way ANOVA test. **p<0.01, *** *p*<0.001.

In order to understand the possible molecular events associated with VA-induced growth arrest in MCF-7 cells, various cell cycle regulatory proteins were examined by western blot analysis. Since VA was shown to induce cell specific G1/S cell cycle arrest, and it is known that MCF-7 cells express wild type p53, it was hypothesized that VA can induce activation of p53, a tumour suppressor gene that plays a vital role in the cell cycle. p53 activation is the most commonly implicated mechanism of G1-phase arrest following drug exposure [11]. Hence, p53, p21^Waf1/Kip1^ (a downstream transcriptional target gene of p53 and a cyclin-dependent kinase inhibitor) and other cell cycle proteins regulating cell cycle progression at the G1/S boundary such as cyclin D1 and cyclin E were detected by western blotting. As depicted in **Figure 3A and 3B**, VA treatment remarkably increased the p53 expression level in a time-dependent pattern in MCF-7 cells. Expectedly, the increased p53 level was correlated with an up-regulation of its transcriptional target gene, p21. The expression levels of cyclin D1 and cyclin E significantly decreased within 12 h of VA treatment; the suppression remained throughout the treatment duration. Together, these results suggest that VA arrested MCF-7 cells in the G1/S phase of the cell cycle through the up-regulation of p53 and p21 genes and the suppression of G1 cell cycle regulators.

By contrast, western blot analysis of VA on the expression of cell cycle regulators in MDA-MB-231 cells revealed that VA played a negligible role in cell cycle progression (**Figure 3C and 3D**). No significant and consistent changes in the expression of p53, p21, cyclin D1 and cyclin E could be clearly observed after exposure to VA for 48 h. These results indicate that the VA-treated MDA-MB-231 cells were not arrested in the G1/S phase as VA failed to activate the cyclin-dependent kinase inhibitor and suppress G1 cell cycle regulators, resulting in no cell cycle arrest in MDA-MB-231 cells.

### VA induced apoptosis in MCF-7 and MDA-MB-231 cells

**Figure 4:**
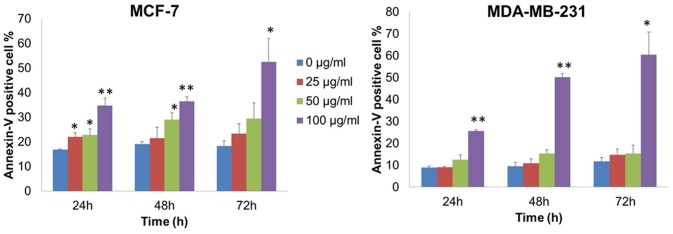
Quantitative analysis of VA-induced apoptosis in MCF-7 and MDA-MB-231 breast cancer cells as assessed by Annexin V-FITC staining assay. Cells were treated with indicated concentrations for 24, 48, and 72-FITC and PI staining. Values shown are means ± S.E.M. of two or three independent experiments. Statistical differences were analyzed with one-way ANOVA test. *p<0.05, **p<0.01.

Since apoptotic cells with hypodiploid DNA content were detected in the sub-G1 phase of the cell cycle, another apoptosis marker, phosphatidylserine exposure was examined by Annexin V-FITC/PI assay using flow cytometry to further investigate if VA could induce apoptosis in the breast cancer cells. It was observed that VA induced apoptosis in MCF-7 cells in a dose- and time-dependent manner (**Figure 4**). After 24 h exposure to VA, increasing doses resulted in an increased proportion of apoptotic cells by more than twofold, from 16% in the control to 35% in the 100 µg/ml VA. Following 48-h and 72-h VA treatment, Annexin V-FITC-stained positive cells increased to 37% and 53% respectively. For MDA-MB-231 cells, apoptotic cells were found to be most pronounced after treatment with the highest concentration of VA (100 µg/ml). The Annexin V-FITC-stained positive cells increased from 9% in the control to 26% at 24 h, from 9% to 50% at 48 h, and from 12% to 60% at 72 h treatment (**Figure 4**). Interestingly, the apoptotic effect induced by VA appeared to be less evident in MDA-MB-231 cells than in the MCF-7 cells, implying the presence of other unknown mechanisms induced by VA in MDA-MB-231 cells. These results thus demonstrate the ability of VA to induce cell death in MCF-7 and MDA-MB-231 cells through apoptosis, though to varying degrees.

### VA induced apoptosis through activation of caspases and alterations in the levels of Bcl-2 family proteins in MCF-7 and MDA-MB-231 cells

**Figure 5:**
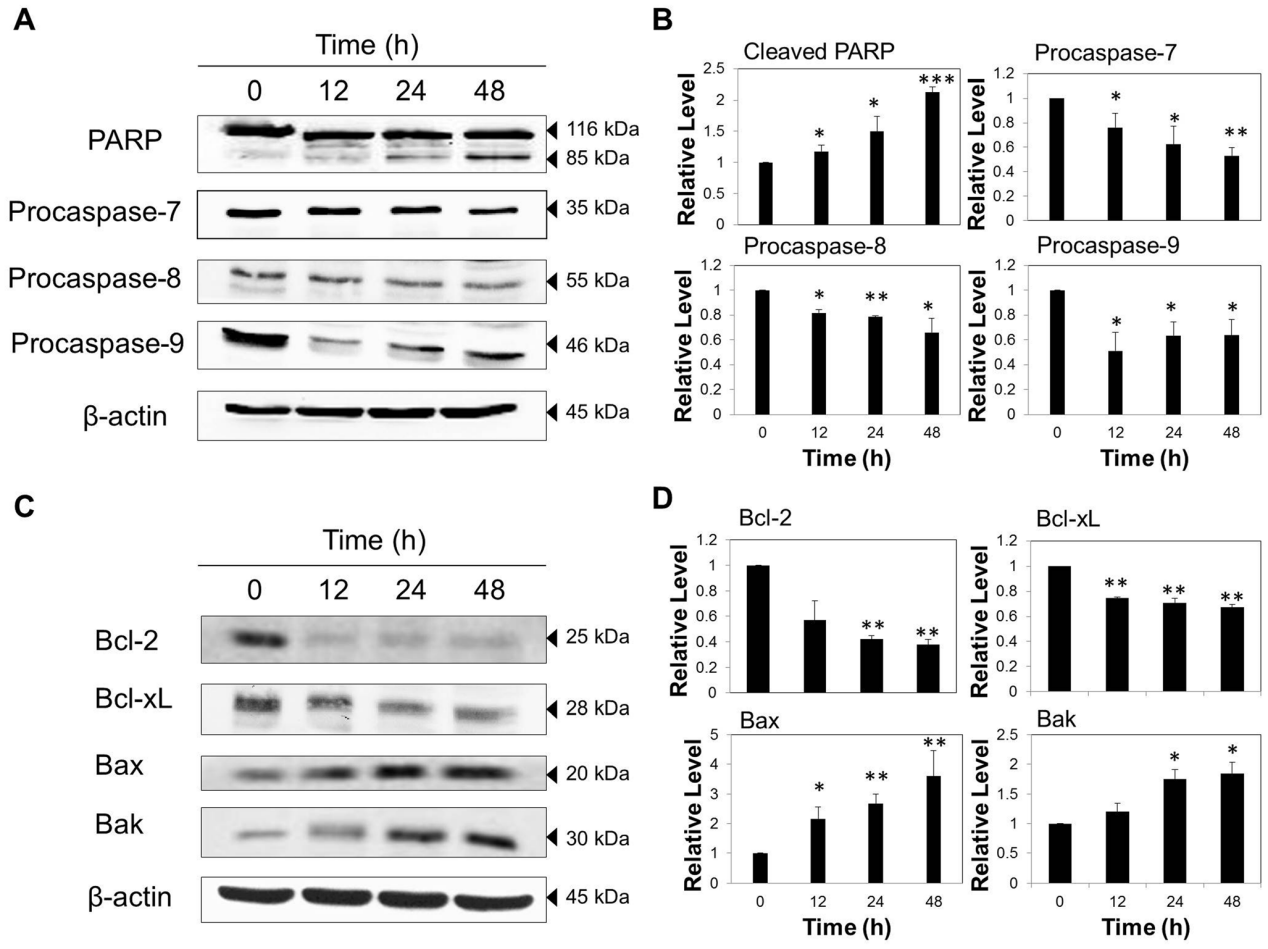
VA induced time-dependent apoptosis in MCF-7 cells. Cells were treated with VA (50 µg/ml) up to 48 h. Whole-cell lysates were resolved on SDS-PAGE gel and probed with the indicated antibodies. β-actin was used as a loading control. (A) Cleavage of PARP and down-regulation of procaspases were detected by western blot. (C) Down-regulation of anti-apoptotic proteins (Bcl-2 and Bcl-xL) and up-regulation of pro-apoptotic proteins (Bax and Bak) were observed by western blot. The data are representative of at least three independent experiments. (B) and (D) Protein quantification of the western blot results shown in (A) and (C) respectively. Protein levels were normalized to the β-actin level and are shown relative to the DMSO-treated control cells (normalized at 1). Statistical differences were analyzed with one-way ANOVA test. *p<0.05, **p<0.01, *** *p*<0.001

To delineate the possible signalling pathways by which VA induced apoptosis in MCF-7 cells, the changes in the expression levels of various apoptosis-regulating proteins such as initiator caspases (caspase-8 and -9), effector caspases (caspase-3 and -7), PARP (poly-ADP ribose polymerase), and Bcl-2 family members (Bcl-2, Bcl-xL, Bax, and Bak) were studied by western blotting. As depicted in **Figure 5A and 5B**, the exposure of MCF-7 cells to VA for 48 h resulted in the down-regulation of procaspase-7, -8, -9, suggesting the cleavage of caspases and hence their activation in both intrinsic and extrinsic apoptotic pathways. While there was a marked decrease in the level of procaspase-9 from 12 h to 24 h, it increased slightly after 48 h exposure. The down-regulation of procaspase-7 and -8 at 12 h was less evident than that of procaspase-9, suggesting that the activation of caspase-9 may be prior to that of caspase-7 and caspase-8. Moreover, VA induced a modest increase in the expression level of cleaved PARP at 24 h, and the increase persisted and became more apparent at 48 h as compared to the untreated control. An activation of caspase-7 induces the proteolytic cleavage of PARP, and since the cleavage of PARP was observed, this indicates that caspase-7 was activated. As PARP is a biochemical marker of cells undergoing apoptosis, the results suggest that the process of apoptosis was triggered.

To further investigate the molecular events that occur upstream of caspase activation and the role of the intrinsic apoptotic pathway in VA–induced apoptosis, it is important to examine the protein levels of Bcl-2 family members, which are the crucial regulators of the intrinsic pathway of apoptosis. Following 48-h treatment, it was apparent that VA-treated MCF-7 cells exhibited a significant decrease in the levels of anti-apoptotic proteins, Bcl-2 and Bcl-xL, concomitant with a marked increase in pro-apoptotic protein levels, Bax and Bak (**Figure 5C and 5D**). The level of Bcl-2 decreased abruptly as early as 12 h after VA treatment while Bcl-xL had a gradual decline across time. The increase in the expression of Bax was more pronounced as compared to that of Bak. This time-dependent up-regulation of Bax/Bcl-2 ratio provides a strong indication that the intrinsic apoptotic pathway may play a role in VA-induced apoptosis in MCF-7 cells.

**Figure 6:**
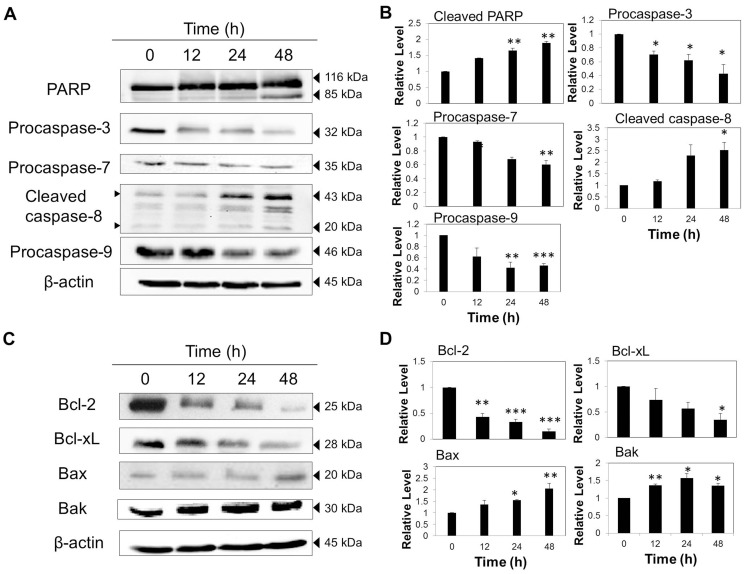
VA induced time-dependent apoptosis in MDA-MB-231 cells. Cells were treated with VA (50 µg/ml) up to 48 h. Whole-cell lysates were resolved on SDS-PAGE gel and probed with the indicated antibodies. β-actin was used as a loading control. (A) Cleavage of PARP and down-regulation of procaspases were detected by western blot. (C) Down-regulation of anti-apoptotic proteins (Bcl-2 and Bcl-xL) and up-regulation of pro-apoptotic proteins (Bax and Bak) were observed by western blot. The data are representative of at least three independent experiments. (B) and (D) Protein quantification of the western blot results shown in (A) and (C) respectively. Protein levels were normalized to the β -actin level and are shown relative to the DMSO-treated control cells (normalized at 1). Statistical differences were analyzed with one-way ANOVA test. *p<0.05, **p<0.01, *** *p*<0.001

Similarly, VA-treated MDA-MB-231 cells demonstrated a significant decrease in the expression levels of procaspase-9 and procaspase-7 in a time-dependent manner and a marked increase in the cleaved caspase-8 level (**Figure 6A and 6B**), suggesting the activation of both extrinsic and intrinsic apoptotic pathways. The absence of caspase-3 in MCF-7 cells has been confirmed by western blotting (data not shown) due to the functional deletion of the CASP-3 gene. However, in MDA-MB-231 cells, procaspase-3 was shown to be significantly down-regulated upon treatment of VA for 48 h, indicating the cleavage of caspase-3, and in turn denotes the activation of caspase-3. Additionally, cleavage of PARP was evident after 48 h of VA exposure, revealing the trigger of apoptosis by caspase-3 activation. These results suggest that the induction of apoptosis by VA in MDA-MB-231 cells was through extrinsic and intrinsic apoptotic pathways. In general, VA exerts its apoptotic effect in MDA-MB-231 cells at a later time point as compared to MCF-7 cells, as most changes in the apoptosis-regulating proteins were more pronounced after 24 h exposure.

Likewise, the expression levels of Bcl-2 family members in MDA-MB-231 cells were also modulated by VA. **Figure 6C and 6D** illustrate the gradual decline in the expression levels of both Bcl-2 and Bcl-xL in a time-dependent pattern while Bak and Bax reflected significant and modest increases respectively, from 12 h to 48 h. These results indicate that the intrinsic apoptotic pathway may be one of the mechanisms of VA-induced apoptosis in MDA-MB-231 cells.

### VA induced caspase-dependent apoptosis in MCF-7 cells

**Figure 7:**
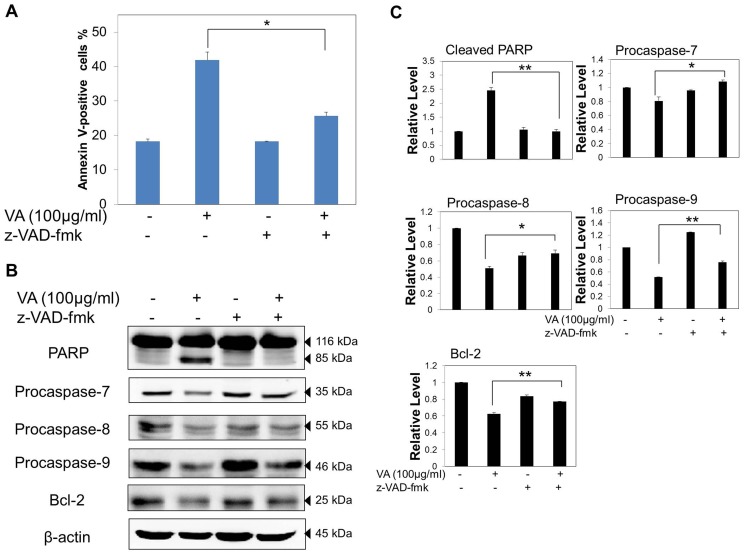
VA induced caspase-dependent apoptosis in MCF-7 human breast cancer cells. General caspase inhibitor, z-VAD-fmk alleviated VA-induced apoptosis. Cells were pre-treated with 20 µM of z-VAD-fmk for 2 h followed by co-incubation with VA for 24 h. (A) Quantitative analysis of apoptotic cells after staining with Annexin V-FITC/PI and analysing by flow cytometry. Values shown are means ± S.E.M. of two independent experiments. *p<0.05. (B) Western blot analysis of PARP, caspases, and Bcl-2 in the presence of z-VAD-fmk. Whole cell lysates were resolved on SDS-PAGE gel and subjected to western blot analysis. The data are representative of two independent experiments. (C) Protein quantification of the western blot results shown in (B). Protein levels were normalized to the β -actin level and are shown relative to the DMSO-treated control cells (normalized at 1). Statistical differences were analyzed with one-way ANOVA test. *p<0.05, **p<0.01

To further confirm the role of caspases in VA-induced apoptosis, a general caspase inhibitor, Z-Val-Ala-Asp-fluoromethylketone (z-VAD-fmk) was utilized in this study. Results from Annexin V-FITC/PI assay revealed that z-VAD-fmk remarkably reduced the VA-induced apoptotic cell numbers in MCF-7 cells, but not to the basal level (**Figure 7A**). This result implies that suppression of caspases inhibited apoptosis induced by VA. Western blotting analysis of the PARP expression level demonstrated that pre-treatment of z-VAD-fmk inhibited VA-mediated apoptosis, as the inhibition of caspase activation prevented cleavage of PARP (**Figure 7B and 7C**). Z-VAD-fmk also reversed the VA-induced Bcl-2 expression level, suggesting that the inhibition of caspases activation also affected the intrinsic apoptotic pathway. Together, these results indicate that VA-induced apoptosis in MCF-7 cells is at least partially caspase-dependent.

### VA induced p53-independent cell cycle arrest and apoptosis in MCF-7 cells

**Figure 8:**
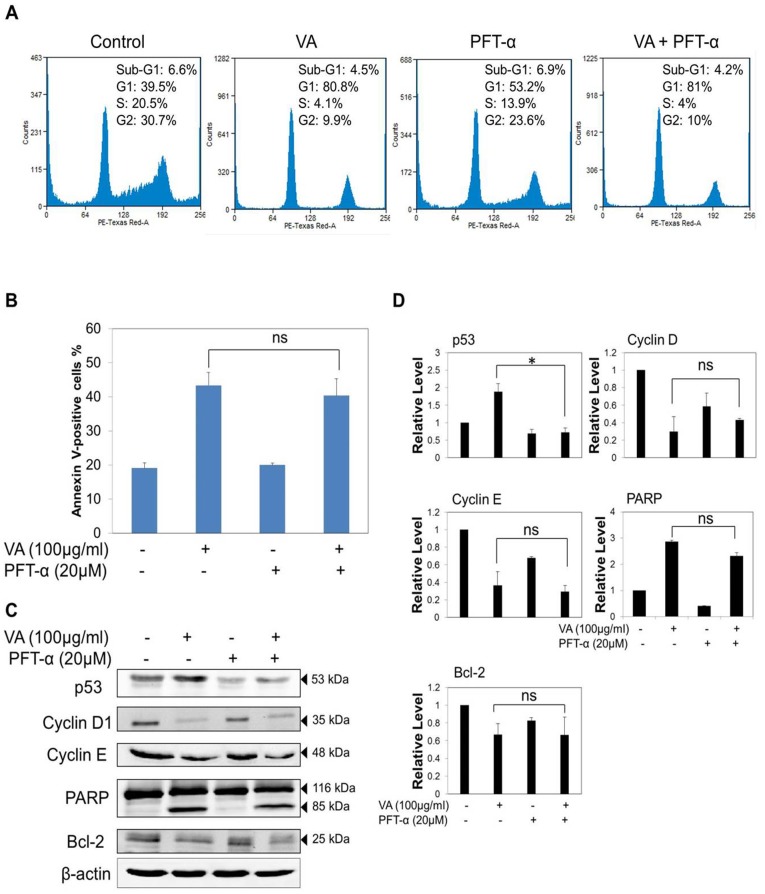
VA induced p53-independent G1/S cell cycle arrest and apoptosis in MCF-7 cells. Cells were pre-treated with 20 µM of pifithrin-α (PFT-α) for 2 h followed by co-incubation with VA for 24 h. (A) Inhibitory effects of PFT-α on cell cycle distribution was analyzed by flow cytometric analysis. Cells were starved for 24 h prior to PFT-α and VA exposure. The data are representative of two independent experiments. (B) Annexin V-FITC/PI assay analysis of the effects of PFT-α on VA-induced apoptosis. Values shown are means ± S.E.M. of two independent experiments. ‘ns’ means non-significant (*p*>0.05). (C) Western blot analysis of the effects of PFT-α on the expression levels of p53, cell cycle regulators and apoptotic-related proteins. The data are representative of three independent experiments. (D) Protein quantification of the western blot results shown in (C). Protein levels were normalized to the β -actin level and are shown relative to the DMSO-treated control cells (normalized at 1). Statistical differences were analyzed with one-way ANOVA test. *p<0.05; ns means non-significant (*p*>0.05).

Based on the results from **Figure 3A and 3B**, it was originally hypothesized that VA-induced cell cycle arrest was dependent on up-regulation of p53 and that the inhibition of p53 would protect cells from VA-induced growth arrest. Hence, the role of p53 in VA-induced cell cycle arrest was verified via a pharmacological approach by using a specific wild type p53 inhibitor, pifithrin-α (PFT-α). PFT-α is a small molecule that binds to the DNA binding domain of p53, thereby inhibiting its transcriptional activity [12]. MCF-7 cells were pre-treated with 20 µM PFT-α (a concentration that did not produce significant toxicity in MCF-7 cells) for 2 h prior to addition of VA (100 µg/ml). PFT-α has been found to enhance cytotoxicity at higher concentrations of more than 30 µM [13]. Unexpectedly, the inhibition of p53 transcriptional activity with PFT-α could not reverse the effect of VA-induced cell cycle arrest as the percentage of cells in the G1 phase remained unchanged (**Figure 8A**). Western blot analysis revealed that pre-treatment with PFT-α decreased the expression level of p53 significantly (**Figure 8C and 8D**), suggesting the inhibition of VA-induced p53 activation by PFT-α. Despite the suppression of p53 expression level, PFT-α could not reverse the decrease of cyclin D1 and cyclin E expression levels induced by VA. Thus, this suggests that VA may partially mediate cell cycle arrest via a p53-independent pathway. However, although our data showed that the pre-treatment with PFT-α could reverse VA-induced p53 expression to the basal level, the treatment did not completely diminish p53 activity. The reduction of the cyclin D1 and cyclin E expression levels may thus not be completely independent of p53.

Moreover, since p53 exerts its tumour suppression effect mainly through the induction of apoptosis, its role in the VA-induced apoptotic pathway was also investigated using PFT-α. As illustrated in **Figure 8B**, PFT-α played a negligible role in the inhibition of VA-induced apoptosis. In addition, western blot analysis of PARP and Bcl-2 expression levels showed that VA-induced apoptosis in MCF-7 cells could not be reversed via the inhibition of p53 transcriptional activity (**Figure 8C and 8D**). Overall, these results indicate that the process of VA-induced apoptosis in MCF-7 cells did not involve a p53 transcriptional dependent pathway.

### VA inhibited ER-α and Akt phosphorylation in MCF-7 and MDA-MB-231 cells

**Figure 9:**
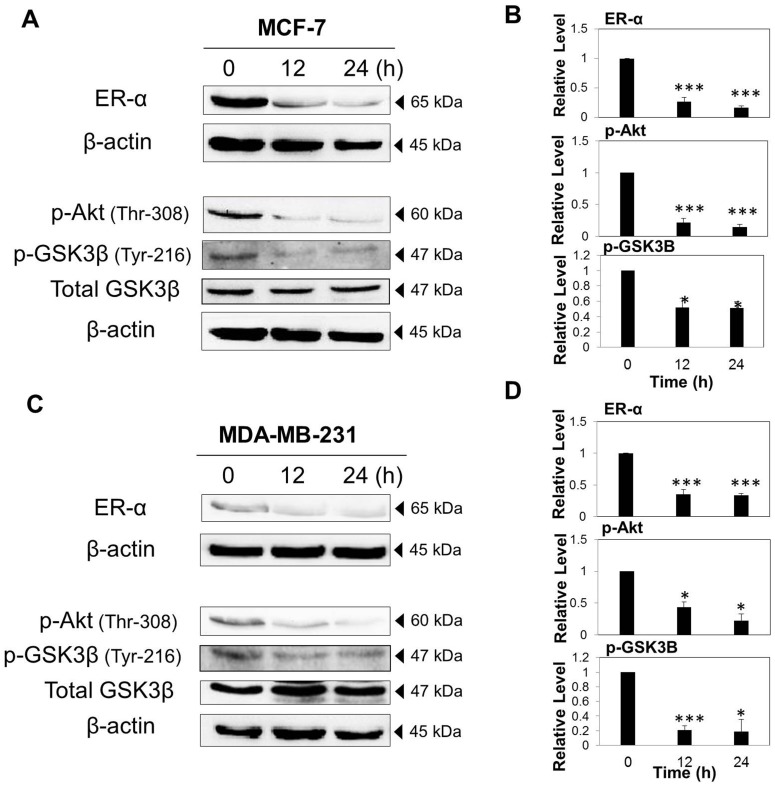
VA inhibited ER-α and the phosphorylation of Akt in MCF-7 and MDA-MB-231 cells. Cells were treated with VA (50 µg/ml) up to 48 h. Whole cell lysates were resolved on SDS-PAGE gel and probed with the indicated antibodies for western blot analysis. β -actin was used as loading control. VA down-regulated the expression of ER-α, p-Akt and p-GSK3 β in (A) MCF-7 cells and (C) MDA-MB-231 cells. The data are representative of three independent experiments. (B) and (D) Protein quantification of the western blot results shown in (A) and (C) respectively. Protein levels were normalized to the β -actin level and are shown relative to the DMSO-treated control cells (normalized at 1). Statistical differences were analyzed with one-way ANOVA test. *p<0.05, *** *p*<0.001

Since ER status is recognized as an important clinical predictor of response to the current breast cancer hormonal therapy [14], it is equally vital to investigate the effect of VA on ER signalling in MCF-7 and MDA-MB-231 cells. As shown in **Figure 9A and 9B**, the exposure of MCF-7 cells to VA for 24 h resulted in the down-regulation of ER-α expression as early as 12 h, and the suppression was even greater at 24 h. This result suggests that VA could be an ER ligand with the ability to inhibit its expression. While MDA-MB-231 cell line has been known to be an ER-negative breast cancer cell line, a very low expression of ER-α can still be detected and VA treatment inhibited the ER-α expression significantly (**Figure 9C and 9D**). These results highlight not only the fact that ER-α is present in the two cell lines (though with different degrees of expression), but also the ability of VA to inhibit its expression even if the expression is minimal, attesting to its sensitivity.

ER affects a wide range of downstream signalling, one of which is the Akt signalling pathway, and thus the effect of VA on Akt was investigated. Western blot analysis showed that VA inhibited the phosphorylation of Akt at Threonine 308 in both MCF-7 (**Figure 9A and 9B**) and MDA-MB-231 cells (**Figure 9C and 9D**), but to a greater extent and longer duration in MCF-7 cells (**Figure 9A and 9B**). This result provides even more evidence that VA can induce cell cycle arrest and apoptosis because the inhibition of Akt leads to anti-survival and anti-proliferation biological effects. One of the downstream effectors of Akt, GSK3β (Glycogen synthase kinase 3β) has a direct effect on the cell cycle. Suppression of Akt activation led to the inhibition of GSK3β phosphorylation at Tyrosine 216 in both MCF-7 (**Figure 9A and 9B**) and MDA-MB-231 cells (**Figure 9C and 9D**), suggesting the possible involvement of Akt pathway in VA-induced cell cycle arrest.

### VA demonstrated synergistic effect with doxorubicin in MCF-7 and MDA-MB-231 cells

**Figure 10:**
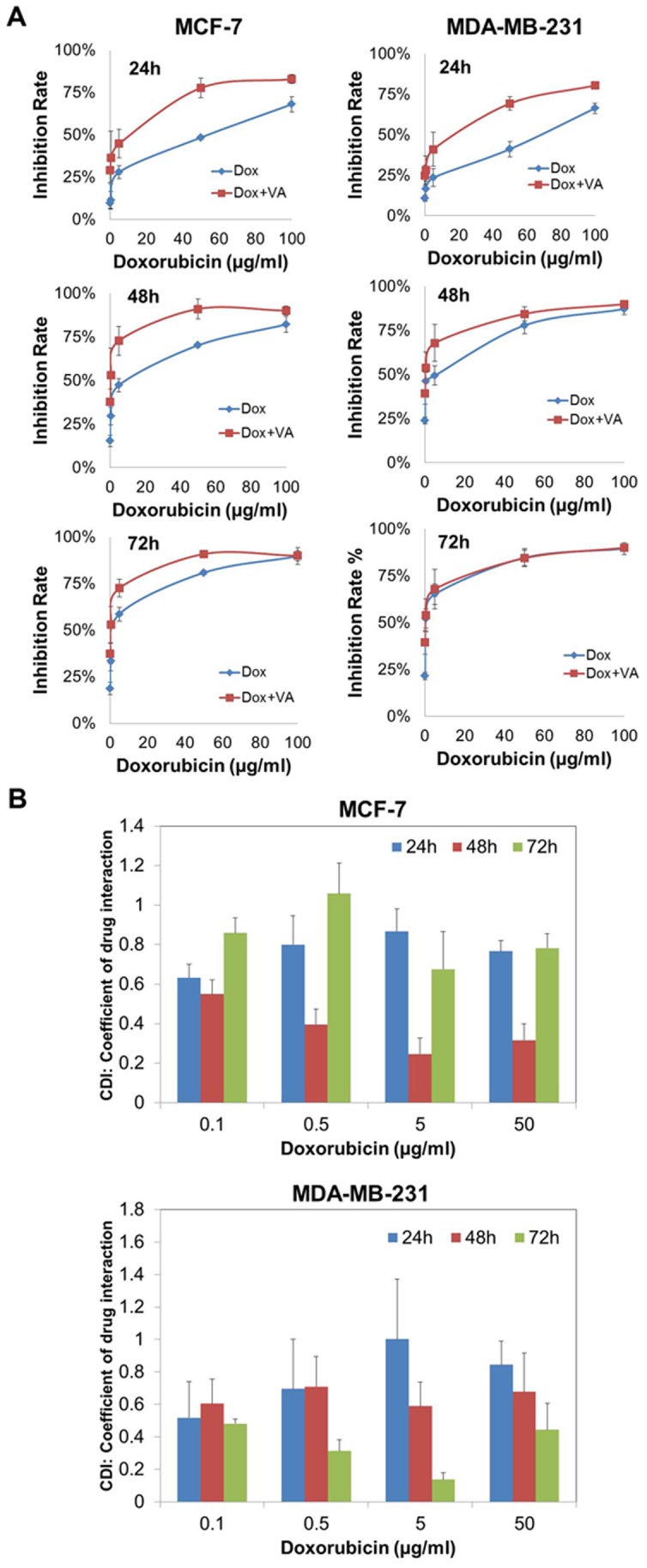
The synergistic effect of VA combined with doxorubicin on the growth of MCF-7 cells and MDA-MB-231 cells. (A) Growth inhibition rate of doxorubicin alone (0.1–100 µg/ml) or in combination with VA (30 µg/ml) after exposure for 24 h, 48 h and 72 h. Inhibitory effect was determined using MTT assay. (B) Coefficient of drug interaction (CDI) values for the combination treatment of doxorubicin with VA (30 µg/ml) on MCF-7 cells. CDI<1 or <0.7 indicate that the drugs are synergistic or significant synergistic respectively. Values shown for all the experiments are means ± S.E.M. of at least three independent experiments. Statistical differences were analyzed with Student's t-test.

Synergistic cell killing without increasing non-specific toxicity is a frequent goal of drug combinations in clinical use. New anticancer agents are usually combined with current chemotherapeutic drugs as a combination therapy to achieve better clinical outcomes. It is therefore worthwhile to investigate the synergistic inhibitory effect of VA and the conventional chemotherapeutic drug, doxorubicin, on MCF-7 and MDA-MB-231 cells by MTT assay. Different concentrations of doxorubicin (0.5 – 100 µg/ml) were combined with 30 µg/ml VA, a concentration that did not lead to significant toxicity in the cells, for 24, 48 and 72 h respectively. The inhibitory effects of doxorubicin alone or combined with 30 µg/ml VA on MCF-7 cells and MDA-MB-231 cells increased in dose- and time-dependent patterns (**Figure 10A**). For instance, in MCF-7 cells, after exposure to an increasing dose of doxorubicin alone for 24 h, the inhibitory rate increased from 9.7% (0.1 µg/ml) to 68.1% (100 µg/ml). As for MDA-MB-231 cells, the rate increased from 10.7% (0.1 µg/ml) to 66.3% (100 µg/ml) at 24 h. It is notable that the combination of doxorubicin and 30 µg/ml VA significantly increased the inhibitory effects on both MCF-7 and MDA-MB-231 cells as compared to doxorubicin alone. In particular, the combined treatment resulted in the increase of the inhibitory rate in MCF-7 cells from 29.2% (0.1 µg/ml) to 82.9% (100 µg/ml) at 24 h, and in MDA-MB-231 cells from 24.8% (0.1 µg/ml) to 80.4% (100 µg/ml) at 24 h. These results suggest that the combined treatment of VA and doxorubicin enhance the cytotoxicity action of each other in MCF-7 and MDA-MB-231 cells.

The synergistic inhibitory effect of drug combinations of VA and doxorubicin were analyzed using the coefficient of drug interaction (CDI) as calculated based on the formula shown in section 2.8. As shown in **Figure 10B**, the combinations of varied concentrations of doxorubicin and 30 µg/ml VA yielded synergistic interactions across a wide range of concentrations and timings (CDI<1). A common trend can be observed in synergistic interactions between MCF-7 cells and MDA-MB-231 cells, which is that the most prominent synergistic effect in MCF-7 cells occurred after 48 h exposure, whereas the effect in MDA-MB-231 cells appeared after exposure for 72 h, indicating the quicker response to VA in MCF-7 cells than MDA-MB-231 cells. For example, while the most prominent synergistic effect across three different time points was 0.25 of CDI when 5 µg/ml of doxorubicin combined with 30 µg/ml VA after exposure of 48 h in MCF-7 cells, the same combination of doses was found to yield the most significant synergistic effect in MDA-MB-231 cells, with CDI value of 0.14 but with a longer time, 72 h. Overall, this study showed that the combination of doxorubicin and VA indeed produces synergism in MCF-7 and MDA-MB-231 cells, laying a foundation for further investigations about the application of VA as a complement to doxorubicin. Another chemotherapeutic drug, namely 5-florouracil, was also tested for its synergistic effect when combined with VA, but no synergistic effect was found (data not shown).

## Discussion

The aims of this study were to determine the anti-cancer effects of VA against human breast cancer cells and its possible mechanisms of action. The present report thus delineates the underlying mechanisms by which VA induces cell cycle arrest and apoptosis in the cancer cells. VA inhibited the proliferation of MCF-7 and MDA-MB-231 cells in a time- and dose-dependent manner. In addition, it caused cell-type specific G1/S growth arrest in MCF-7 cells through the suppression of the expression of cyclin D1 and cyclin E, but via a p53-independent pathway. Furthermore, VA increased the number of apoptotic cells as demonstrated by the Annexin V-FITC/PI assay. It inhibited the expression of anti-apoptotic Bcl-2 family members such as Bcl-xL and Bcl-2, and activated pro-apoptotic proteins like Bax and Bak. It further activated caspase-8 and caspase-9 which subsequently induced caspase-3 and/or caspase-7 activation, resulting in PARP cleavage. Crucially, VA blocked ER-α expression, Akt and GSK3β phosphorylation in both MCF-7 and MDA-MB-231 cells. It was also identified that the inhibition of caspases by the general caspase inhibitor, z-VAD-fmk, blocked VA-induced apoptosis in MCF-7 cells, but the cells were not spared from VA-induced apoptosis when p53 transcriptional activity was suppressed by PFT-α. Finally, synergism was observed when VA was combined with doxorubicin.

The results suggest that VA induced a strong cytotoxic effect on MCF-7 and MDA-MB-231 cells by blocking their proliferation, with IC_50_ values of ≤100 µg/ml, respectively. The IC_50_ values of water-soluble VA extract in MCF-7 cells reported in other studies demonstrate enormous discrepancy, ranging from 5.6 µg/ml to 1000 µg/ml [2], [15], [16]. As plant extract comprises several different active biological compounds, it is likely that the inconsistency of VA potencies is due to batch variation, in which certain batches may have higher activity than others. Despite the inconsistency of IC_50_ values, the present study nevertheless confirms the growth inhibitory effect of VA in MCF-7 cells. It was also showed, for the first time, that VA demonstrated its ability to inhibit proliferation of MDA-MB-231 cells at a potency that is slightly lower than that in MCF-7 cells. These results suggest that VA is able to inhibit the proliferation of human breast cancer cells regardless of the cell type, highlighting its broad-spectrum cytotoxic effects.

Despite the fact that VA inhibited growth of both MCF-7 and MDA-MB-231 cells, it blocked proliferation by inducing cell cycle arrest at G1/S phase in MCF-7, but not in MDA-MB-231 cells. This selective effect of VA thus prompted an analysis of the differential changes in cell cycle regulatory proteins induced by VA. Western blot analysis has revealed that VA induced activation of p53 and p21 while suppressing the expression of cyclin D1 and cyclin E in MCF-7 cells. This provides strong evidence that VA arrested MCF-7 cells in the G1 phase, thereby inhibiting their progression to the S phase. However, there were no significant changes in the expression of p53, p21, cyclin D1 and cyclin E after VA treatment in MDA-MB-231 cells. Owing to this observation, this study made the postulation that this selective effect of VA on cell cycle arrest in MCF-7 cells could be due to the difference in p53 status between the two cell lines. Unexpectedly, the partial suppression of p53 transcriptional activity by PFT-α failed to reverse the cell cycle arrest induced by VA in the MCF-7 cells as shown in **Figure 8**. Thus, it is possible that the VA-induced cell cycle arrest in MCF-7 cells may be independent of p53.

Although p53 may not responsible for the VA-induced cell cycle arrest in our study, the increased expression as detected by western blotting is believed to be due to the DNA damage induced by VA. This postulation can be supported by a report in which VA was shown to induce minimal DNA damage in MCF-7 cells as assessed by alkaline single cell gel electrophoresis (Comet) assay [2]. Since it is widely agreed that DNA-damage response is integral to the actions of p53 as a tumour suppressor, it is believed that VA can induce DNA damage in MCF-7 cells, which in turn activates p53 due to the release from Mdm2, and subsequently triggers various downstream effects.

The results from this study indicated that VA induced apoptosis in MCF-7 and MDA-MB-231 cells as measured by flow cytometry. At the molecular level, VA treatment down-regulated the expression of Bcl-2 and Bcl-xL and up-regulated the expression of Bax and Bak in MCF-7 and MDA-MB-231 cells. This suggests the inhibition of the anti-apoptotic signal and the induction of the pro-apoptotic signal, thereby affecting the mitochondrial permeability. Given that the results showed the activation of caspase-9, it is possible that VA stimulated the release of cytochrome c, which in turn facilitated the formation of apoptosome complexes. The results also showed the activation of caspase-3 and caspase-7 in MDA-MB-231 cells, and caspase-7 in MCF-7 cells, after which both were followed by PARP cleavage. One possible mechanism by which VA induces apoptosis is thus through modulating the expression of Bcl-2 family members to affect membrane permeability, which in turn results in the sequential activation of caspase-9, caspase-3 and/or -7 and ultimately, PARP cleavage. These observations are in agreement with a report in which VA was shown to alter the cell membrane permeability in MCF-7 cells [16]. The results also indicate that VA induces apoptosis partly through the activation of caspase-8 in MCF-7 and MDA-MB-231 cells. However, the mechanism by which VA activates caspase-8 remains to be elucidated. Nevertheless, this study suggests another possible mode of action by which VA induces apoptosis through the induction of the extrinsic apoptotic pathway. It is still unclear whether VA-induced activation of caspase-8 can cleave Bid, which is a BH3-only pro-apoptotic protein that can initiate the mitochondrial pathway. Further research on the expression of Bid is required to determine if cross-talk between the mitochondrial intrinsic pathway and the death-receptor-mediated extrinsic pathway does exist in VA-induced apoptosis.

Although caspase may be a necessary factor in the execution of programmed cell death, the process of caspase activation is not the sole factor in determining the triggering of apoptosis. Some studies have reported the model of caspase-independent cell death in different cell types, such as Jurkat, MCF-7 and Hela cells [17]-[19]. Therefore, it is important to determine if VA-induced apoptosis can still occur in the presence of the caspase inhibitor, z-VAD-fmk. Z-VAD-fmk is a cell-permeable tripeptide inhibitor which contains aspartate residue and fmk group, mimicking the cleavage site of caspase and forming a covalent inhibitor/enzyme complex [20]. It works by binding irreversibly to the catalytic site of caspases [20]. The results from this study highlight the important role of caspases in VA-induced apoptosis in MCF-7 cells because the inhibition of caspase activity by z-VAD-fmk abolished the PARP cleavage, suppressing the overall apoptosis induced by VA. It is thus possible that VA inhibits the growth of MCF-7 cells through the induction of caspase-dependent apoptosis.

By contrast, the results showed that VA-induced apoptosis in MCF-7 cells was not mediated through a p53-dependent pathway. For the past three decades, p53 has been the subject of intense research interest. The p53 tumour suppressor has been termed ‘the guardian of the genome’ because of its pivotal role in safeguarding the integrity of genetic information in response to various genotoxic injuries [21], [22]. Besides the previously mentioned role of suppressing growth arrest, the induction of apoptosis is one of the central activities by which p53 exerts its tumour-suppressing function. It has been widely known that p53, as a transcription factor, promotes apoptosis through the transcription of its target genes such as Bcl-2 family members. However, an increasing number of studies has shown the existence of a transcription-independent mechanism – i.e. a direct localization of p53 to the mitochondria, such that p53 can interact directly with Bcl-2 or Bcl-xL to promote apoptosis [22], [23]. Thus, it is of paramount importance to determine whether p53 plays a p53 transcription-dependent or independent role in the VA-induced apoptosis. The results obtained from the study showed that blocking the p53 transcriptional activity in MCF-7 cells by PFT-α could not reverse the VA-induced apoptosis as indicated by Annexin V-FITC/PI assay and western blot analysis. It is likely that VA-induced apoptosis in MCF-7 cells may not occur via a p53 transcription-dependent mechanism. However, further research is required to determine whether it is a p53 transcription-independent mechanism or a pathway that is independent of p53 altogether, by using pifithrin-mu (PFT-μ) in addition to PFT-α. PFT-μ works by reducing the binding affinity of p53 to Bcl-2 and Bcl-xL, thereby inhibiting its binding to mitochondria, but without affecting its transactivation activity [12]. It can help to determine if the apoptosis is independent of p53 transcriptional activity. If both PFT-α and PFT-μ fail to reverse the VA-induced apoptosis, it would mean that the VA-induced apoptosis is independent of p53 altogether.

Hormone receptor status has been recognised as the most important prognostic and predictive factor for response to hormonal therapy [24]. As ER status is used as a determinant factor for the current breast cancer treatment [14], agents that can compromise ER signalling promise to be clinically important therapeutic drugs. ER-α is one of the isoforms of ER, which acts as a transcription factor to initiate the transcription of specific target genes upon activation by estrogen [25]. A crucial finding from this study was that besides modulating the expression of apoptosis-regulating molecules, VA also mediates its effects through down-regulation of ER-α expression in MCF-7 cells. Since around 70% of diagnosed breast cancers are ER-positive which express ER-α in particular, the ability of VA to inhibit ER-α expression suggests the potential clinical significance of VA. Although it is widely known that MDA-MB-231 is an ER-negative breast cancer cell line, western blot analysis has revealed a basal level of ER expression. This observation can be supported by the studies conducted by Ford *et al*. [26] who showed that both MCF-7 and MDA-MB-231 cell lines express ER-α and ER-β using flow cytometry, reverse transcription-polymerase chain reaction (RT-PCR) and western blot analysis [26]. Despite the low expression of ER-α in MDA-MB-231 cells, VA was able to reduce its expression, suggesting its high sensitivity towards ER-α. These results provide the basis for future research to further elucidate the potential application of VA as an anti-estrogenic therapeutic agent.

One of the downstream signalling pathways of ER is Akt, which is the master regulator of cell growth and is closely linked to cell survival [27], [28]. The results from this study demonstrate the ability of VA to inhibit the phosphorylation of Akt at Threonine 308 residue, implying the suppression of cell survival and proliferation signals in both MCF-7 and MDA-MB-231 cells. Inhibition of Akt activation is associated with a pro-apoptotic effect by induction of the Bad pro-apoptotic proteins of the Bcl-2 family, leading to apoptosis [28]. Furthermore, Akt inhibition led to the suppression of phosphorylation of GSK3β, which targets cyclin D1 for proteasomal degradation, resulting in cell cycle arrest in the G1 phase [27]. Hence, Akt signalling pathway may be one of the mechanisms of VA in the induction of cell cycle arrest and apoptosis. Other upstream kinases such as PDK1 and PI3K, as well as downstream proteins like JNK, are to be further studied in order to elucidate the actions of VA fully.

There has been growing interest in combination therapy as it induces a greater effect in the improvement of patients' survival [29]. Since cancer is the result of the accumulation of numerous mutations, it is rational to combine two or more drugs with different mechanisms of action to increase cell killing. VA was thus combined with a current chemotherapeutic alkylating agent, doxorubicin, to determine their synergistic effect in human breast cancer cells. Doxorubicin exerts its effects by intercalating base pairs between DNA, thereby inhibiting both DNA and RNA synthesis. In addition, it mediates its main cytotoxic action through inhibiting the activity of topoisomerase II, which is an enzyme responsible for the uncoiling of DNA [30]. These two different mechanisms of action result in DNA disruption that eventually leads to cell death. However, it has been found to be associated with adverse events such as increased risk of bleeding and infection, loss of appetite, cardiac damage and heart failure. In this study, a synergistic effect was observed when VA was combined with doxorubicin in both MCF and MDA-MB-231 cells. This suggests that the combination of VA and doxorubicin at certain concentrations may ameliorate the side effects of doxorubicin treatment. Importantly, the effect of doxorubicin is cell cycle non-specific while that of VA is believed to be G1/S phase-specific. This difference in the mechanism of action allows for more attacks at multiple phases of the cell cycle to accelerate the treatment process, possibly preventing resistance from occurring. Therefore, the synergistic effect was more significant in MCF-7 than MDA-MB-231 cells because VA induces G1/S cell cycle arrest only in MCF-7 cells, which results in more attacks as compared to that of MDA-MB-231 cells. Moreover, different mechanisms of action limit the overlapping toxicities, improving the results of the overall treatment. Hence, these results suggest that VA can act as a complement to current treatment. It is therefore worthwhile to further examine the mechanisms of this synergism so as to evaluate the reasonable applications of VA in human breast cancer treatment.

## Conclusion

In conclusion, VA shows anti-cancer effects in MCF-7 and MDA-MB-231 cells. The effect was mediated through the inhibition of cell proliferation of the breast cancer cells. A novel finding was that the underlying mechanisms of this growth inhibition induced by VA involved the suppression of ER-α and the phosphorylation of Akt, stimulation of cell-specific G1/S cell cycle arrest and the induction of apoptosis through both extrinsic and intrinsic apoptotic pathways. In addition, the VA-induced apoptosis in MCF-7 cells is likely to be caspase-dependent and not p53 transcription-dependent, while the cell cycle arrest is independent of p53. VA also exhibited synergism when combined with doxorubicin, suggesting that it can complement current chemotherapeutic treatment. By detailing the complex mechanisms involved in breast cancer cells, this study confirms the hypothesis, demonstrating the potential applications of VA as an anti-cancer drug and thus paving the way for further research on VA in the field of anti-cancer drug discovery.
